# Biomechanical Evaluation of the Effect of Minimally Invasive Spine Surgery Compared with Traditional Approaches in Lifting Tasks

**DOI:** 10.3389/fbioe.2021.724854

**Published:** 2021-10-18

**Authors:** John Rasmussen, Kristoffer Iversen, Bjørn Keller Engelund, Sten Rasmussen

**Affiliations:** ^1^ Department of Materials and Production, Aalborg University, Aalborg, Denmark; ^2^ AnyBody Technology A/S, Aalborg, Denmark; ^3^ Department of Clinical Medicine, Aalborg University and Aalborg University Hospital, Aalborg, Denmark.

**Keywords:** spine fusion, biomechanics, surgery, simulation, joint loads, muscles

## Abstract

Fusion of spinal vertebrae can be accomplished by different surgical approaches. We investigated Traditional Open Spine Surgery (TOSS) versus Minimally Invasive Spine Surgery (MISS). While TOSS sacrifices spine muscles originating or inserting on the affected vertebrae, MISS seeks to minimize the approach-related morbidity and preserve the tendon attachments of the muscles in the area. We captured 3-D motions of the full body of one healthy subject performing a variety of 10 kg box lifting operations representing activities-of-daily-living that are likely to challenge the spine biomechanically. The motion data were transferred to a full-body biomechanical model with a detailed representation of the biomechanics of the spine, and simulations of the internal spine loads and muscle forces were performed under a baseline configuration and muscle configurations typical for TOSS respectively MISS for the cases of L3/L4, L4/L5, L5/S1, L4/S1 and L3/L5 fusions. The computational model was then used to investigate the biomechanical differences between surgeries. The simulations revealed that joint reaction forces are more affected by both surgical approaches for lateral lifting motions than for sagittal plane motions, and there are indications that individuals with fused joints, regardless of the approach, should be particularly careful with asymmetrical lifts. The MISS and TOSS approaches shift the average loads of different muscle groups in different ways. TOSS generally leads to higher post-operative muscle loads than MISS in the investigated cases, but the differences are smaller than could be expected, given the differences of surgical technique.

## Introduction

Lumbar spinal fusion is a surgical procedure, where two or more of the spinal vertebrae are fused by means of mechanical devices and bone grafts. The indications include a variety of degenerative lumbar spinal diseases. Mobbs et al. (2015) provided a comprehensive review of evidence, indications and surgical approaches.

Presuming that the recovered patient will resume activities of daily living, the motion that previously took place between the fused vertebrae will be redistributed among adjacent spinal joints, which therefore will sustain increased articulation to accommodate the same overall motion of the lumbar spine. The relationship between articulation and net joint reaction is not immediately obvious, but a positive correlation between the two has been hypothesized, and larger articulation with high certainty will cause higher material strain in the disk, and there is clinical evidence for the possibility of adjacent degeneration (Nagata et al., 1993; Aota et al., 1995; Chow et al., 1996; Guigui et al., 1997; Hambly et al., 1998; Etebar and Cahill, 1999; Kumar et al., 2001). On the other hand, the fused joint will transfer moments that were previously balanced by muscles. Thus, the fusion is likely to redistribute the loads on muscles and joints in the region depending on surgical approaches, which might therefore affect the health and longevity of the operated spine.

We shall refer in the following to Traditional Open Spine Surgery with a posterior approach as TOSS. In this approach, access to the affected vertebrae involves resection of a major part of the musculature surrounding the site. Fascicles of the spinal musculature, such as m. erector spinae and m. multifidus that originate or insert on the fused bones, are generally sacrificed, and the same is often the case for fascicles of m. multifidus that cross the site at oblique angles, because they cannot be displaced sufficiently during the surgery. In the presence of a fused, rigid connection between the formerly articulating vertebrae, which supports the joint moments that were previously balanced by muscle actions, it is tempting to think that the local musculature is redundant and that its resection has little or no consequence. However, the spinal muscle configuration is complex with a multitude of fascicles spanning single or multiple joints to articulate and stabilize the spinal column in a statically indeterminate system (Hansen et al., 2006). It is therefore likely that resection of the local muscles has consequences beyond the site.

Minimally Invasive Spine Surgery (MISS) has gained popularity in the past decades (McAfee et al., 2010; Härtl, 2020), based on the reasoning that trauma minimization is generally beneficial for the patient (Kim, 2010), especially since traditional open spine surgery (TOSS) has several reported drawbacks including blood loss, muscle pain and infection risk. Minimally invasive insertion systems are designed to minimize the approach-related morbidity of traditional lumbar pedicle fixation. Depending on the surgical technique, MISS allows for an almost complete preservation of the local musculature.

The consequences of MISS versus TOSS can be assessed retrospectively between patient populations. Favorable results regarding morbidity and infection (Altshuler et al., 2021), readmission and reoperation (Altshuler et al., 2020), and perioperative outcome (Goldstein et al., 2016) for MISS have been reported (Kim et al., 2005). In particular, reduction of surgical trauma in MISS seems obvious and has been confirmed (Stevens et al., 2006). However, reduction of fat infiltration in muscles post-surgery was also investigated and fell below statistical significance (Min et al., 2009), and meta studies (Fourney et al., 2010) failed to show reduction of complications in MISS versus TOSS. Thus, clinical evidence for the biomechanical advantage of MISS over TOSS remains somewhat inconclusive.

The aforementioned clinical studies do not have the resolution to distinguish between the details of the surgery and conditions in the individual patients, and statistics offer little to the causality of observed complications for each patient. Consequently, computer models have been used to make in-silico comparison of TOSS versus MISS. Bresnahan et al. (2010) used a computer model of nominal spine flexion and lateral flexion to confirm the dependency of post-operative muscle activity on the surgical technique in L3/L4 and L4/L5 fusion, and Malakoutian et al. (2016) computed that muscle damage typical of TOSS increases compression loads in adjacent joints in an upright posture. Benditz et al. (2018) simulated the influence of different sagittal alignments in standing postures. Localized tissue models based on finite element analysis (Rijsbergen et al., 2018) have simulated the resulting process of disk degeneration. They draw upon the advantage of detailed geometrical and material description but typically have the disadvantage of absence of simulation of muscle actions, which leaves them to investigate nominal loads. Park et al. (2015) used a finite element model to investigate tissue loads in nine single- and multi-joint fusions under nominal follower loads and moments. Previous computer models have therefore added to the knowledge in the field, but they cover either relatively few fusion sites and/or idealized load cases.

Musculoskeletal models with active muscles as well as experimental techniques to measure human motions have evolved since the aforementioned works in terms of anatomical detail and experimental accessibility. They enable systematic investigation of combinations of real-life load cases and surgical approaches. Computer models also offer the opportunity to investigate all-things-equal situations, where the influence of specific parameters can be computed in the absence of measurement inaccuracies and inter-subject variation. The aim of this paper is therefore to exploit new modeling opportunities to investigate the biomechanical advantages and disadvantages of MISS versus TOSS.

## Methods

**FIGURE 1:**
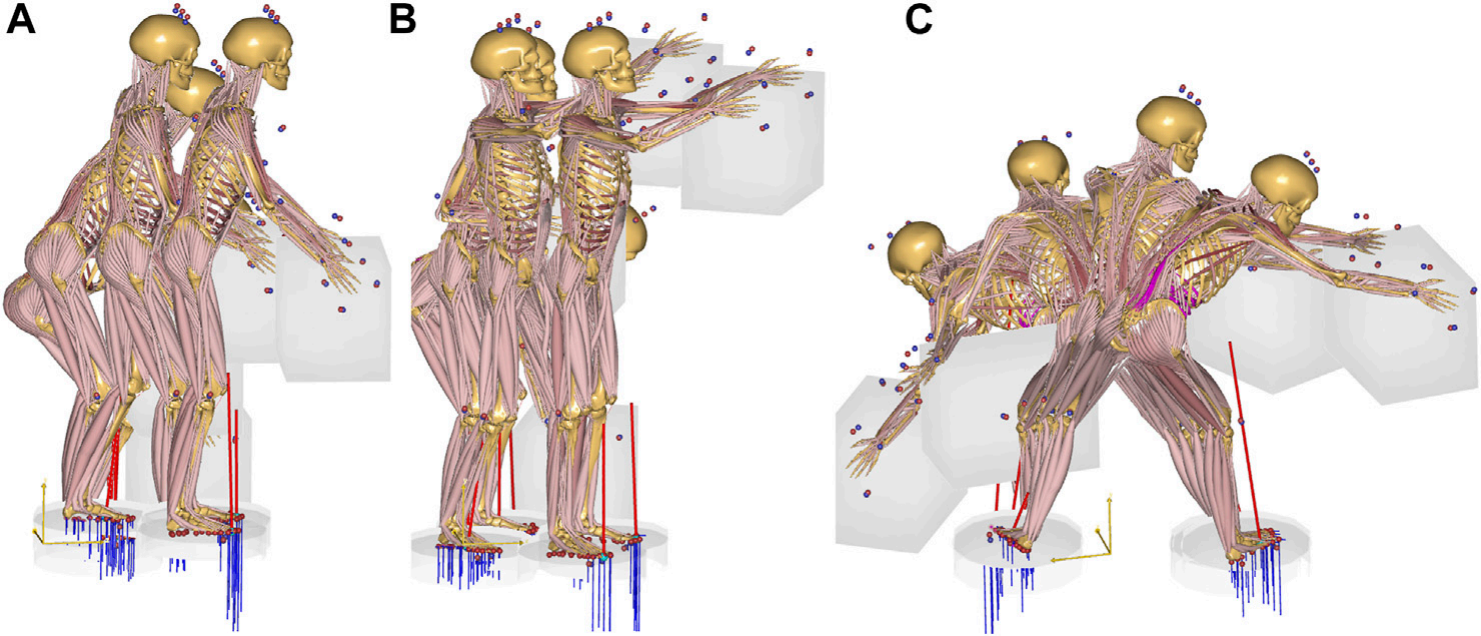
The three box lifting cases. **(A)**: from the floor to 59 cm height. **(B)**: from the floor to 158 cm height. **(C)**: laterally from the floor to 59 cm height.

A single, healthy subject (male, age 29, stature 1.89 m, body weight 82 kg) was recruited for the data collection and signed an informed consent form. The subject lifted boxes weighing 10 kg from the floor to two different heights of (A) 59 cm and (B) 158 cm respectively in a sagittal plane motion, and subsequently (C) from the floor to 59 cm height in a movement from left to right. The test subject was instructed to perform the task naturally and with a technique of his own choice. Before recording the motion, the subject had the opportunity to perform familiarization trials. The three motions, A, B and C, are illustrated in Figure 1.

The motions were recorded with the Xsens Awinda system (Xsens Technologies B.V., Enschede, Netherlands). This is a wearable technology based on inertial measurement units and sensor fusion (Koning et al., 2015), and its suitability for recording musculoskeletal model input has been verified previously (Karatsidis et al., 2018). The sensor positions are on the feet, the lower legs, the upper legs, the pelvis, the sternum, the shoulders, the upper arms, the forearms, the hands and the head. The motions were transferred via a BVH file to the AnyBody Modeling System version 7.3 (AnyBody Technology A/S, Aalborg, Denmark) (Damsgaard et al., 2006). The baseline model was the AnyScript Managed Model repository version 2.3.1 comprising lower extremities, pelvis and lumbar spine, a rigid thoracic spine and rib cage segment, an articulated cervical spine, shoulder complex, upper arms, forearms and rigid hand segments. The model comprises about 1,000 individually activated muscle fascicles. Muscle fascicles are modeled with individual cross sectional areas representing their strength, but the model does not take activation and contraction dynamics into account and its validity is therefore limited to relatively slow and voluntary movements. The model used inverse dynamics and solved for individual muscle forces with a quadratic recruitment criterion.

The lumbar spine model (de Zee et al., 2007) contains the lumbar vertebrae, the sacrum and the pelvis. The disk connections are idealized as spherical joints in the baseline, non-fused condition. The model comprises a total of 178 spinal muscle fascicles distributed over the groups: multifidi, erector spinae, psoas major, quadratus lumborum, semispinalis and spinalis. The model also comprises the abdominal musculature and its connection with the intra-abdominal pressure, which works to extend the lumbar spine as necessary. Scaling of the model to subject-specific dimensions happens on the segment level in response to the processing of the kinematics data, and segment inertial parameters are similarly scaled (Lund et al., 2015). The muscle strengths are scaled according to the BMI using the length-mass-fat scaling law (Rasmussen et al., 2005). The entire musculoskeletal model is continuously updated and published (Lund et al., 2020).

General validation of musculoskeletal models is difficult for a variety of reasons (Lund et al., 2012). For the case of the spine, intradiscal pressures in the intact structure and detailed joint force information from instrumented implants in operated structures have been obtained experimentally and were reviewed in detail by Dreischarf et al. (2016). They lend themselves to comparison with simulated values, and several independent research groups have corroborated the lumbar spine model used in this study (Han et al., 2012; Arshad et al., 2016; Bassani et al., 2017, 2020).

Analysis was performed on a baseline model representing the intact body, in single joint fusions of L3/L4, L4/L5, L5/S1, and in multiple joint fusions of L4/S1 and L3/L5, respectively, in MISS and TOSS configurations, resulting in a total of 33 combinations of analysis. In the MISS configurations, the musculature was intact, and the affected joints were fused to disable mutual motion and allow transfer of force and moment across them, i.e. the fused joints were changed from spherical to rigid joint assumptions and transferred any necessary moment across the fusion without the need for muscle actions. Except from the fusions, the spine model does not contain passive stiffness, i.e. all joint moments are balanced by muscle forces. The spine model’s kinematic rhythm (Hansen et al., 2006) is mathematically equivalent to a movement distribution between the joints according to stiffness, i.e. as if the spine were a discretized elastic beam. This method was proposed by Stokes et al. (2002) based on *in-vitro* measurements of spine deflection. In the current, inverse dynamics model, the kinematics is resolved before kinetics, and the elastic beam assumption leads to a third-order polynomial, spatial spline shape, whose continuous deflection is collected in the discrete joints as flexion/extension, lateral flexion and axial rotation respectively. The third order polynomial for each of these articulations has four unknown coefficients, which are resolved from the four conditions of positional and slope continuity over the connections between the sacrum and the pelvis and T12/L1, respectively. This functional relationship between articulations enters the kinematic problem as constraints. In the presence of rigid, fused joint(s), this constraint set is augmented by high-weight conditions of no articulation between the fused vertebrae, and the resulting over-constrained system is solved by the method of Andersen et al. (2009). The consequence is that the previous articulations of fused joints will transfer to the remaining non-fused joints, which will behave as if the non-fused sections of the spine were discretized elastic beams. In the TOSS scenarios, the joints were also fused, and sacrificed muscle fascicles were removed from the model, leading to redistribution of their force contributions between the remaining muscles according to the recruitment criterion. Thus, joint kinematics were identical for the MISS and TOSS cases, but MISS and TOSS kinematics were different from the baseline case. The muscle configuration was identical for the baseline and MISS cases, and different for each TOSS case.

**FIGURE 2:**
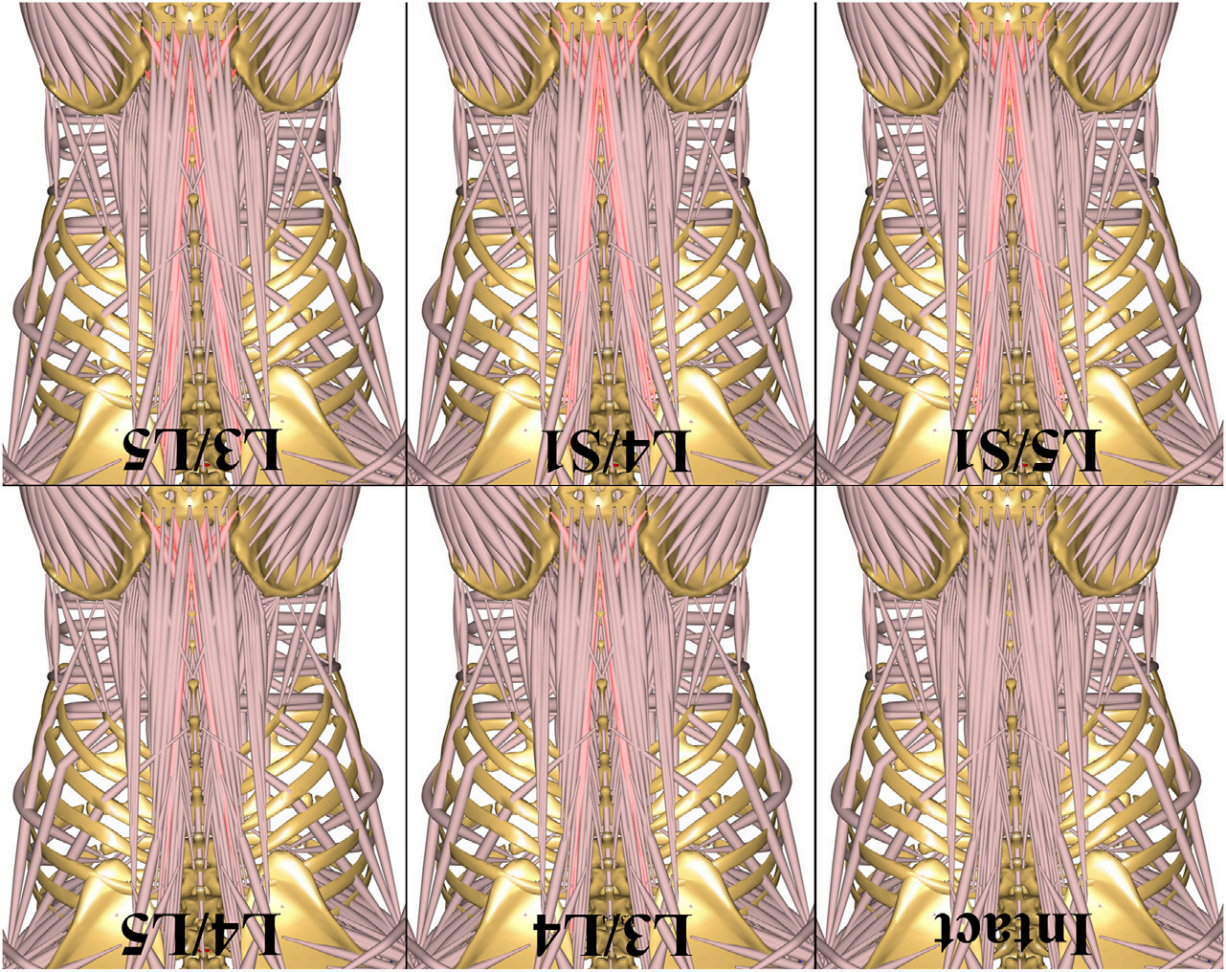
TOSS muscle configurations for different fusions. The resected muscle fascicles for each case are highlighted and concisely listed in Table 1.

Removal of muscle fascicles for each TOSS case was based on the surgical experience of the fourth author and performed interactively in a simulated “virtual surgery” performed on the 3-D graphical representation provided by the AnyBody Modeling System. The intact and resected muscle configurations are illustrated in Figure 2, and the resected muscle fascicles for each case are listed in Table 1, referring to the systematic naming conventions of the baseline model (Hansen et al., 2006; de Zee et al., 2007; Lund et al., 2020).

**TABLE 1:** Resected fascicles of m. erector spinae and m. multifidus in TOSS at each lumbar fusion level. The systematic fascicle names refer to origin and insertion points in the published model (Lund et al., 2020). The mentioned fascicles are resected symmetrically on both sides.

	Lumbar fusion levels				
**Muscle group**	**L3/L4**	**L4/L5**	**L5/S1**	**L4/S1**	***L3/L5***
*Erector Spinae*	LTptT6S1 LTptT5L5	LTptT6S1	LTptT8S3 LTptT7S2 LTptT6S1	LTptT8S3 LTptT7S2 LTptT6S1 LTptT5L5	LTptT6S1 LTptT5L5 LTptT4L4
*Multifidi*	MFtsL3Ligament MFdL4S1 MFmL3S1 MFdL3L5	MFtsL3Ligament MFmL4Sacrum MFtsL4Sacrum MFmL5Sacrum MFdL5S1	MFtsL4Sacrum MFtsL5Sacrum MFmL4Sacrum MFmL5Sacrum MFdL5S1	MFtsL4Sacrum MFtsL5Sacrum MFmL4Sacrum MFmL5Sacrum MFdL5S1 MFtsL3Ligament MFdL4S1	MFtsL4Sacrum MFtsL3Ligament MFmL4Sacrum MFdL5S1 MFtstL2SIPS MFtsL2S1 MFtsL2L5MFmL3S1 MFdL3L5 MFdL4S1MFmL5Sacrum

We report resultant reaction forces, i.e. the norm of the force vector, across the joints for the entire movement, to assess how different surgical combinations affect the loads on the spinal disks in different postures during the lifts. Muscle activity in the following means the percentage force to assumed isometric strength in a given muscle fascicle. To investigate the redistribution of loads between the muscle groups resulting from different fusions and resections, we computed the activity of each muscle group as the average over its fascicles. For each of the resulting average group activity curves, we found the maximum over the movement and computed the shift in percent compared to the baseline case. Resected muscles were completely removed from the model and therefore not included in the average.

## Results

This section presents resulting joint reaction forces, which acted on the spinal disks, and changes of muscle group activities in response to fusion and approach combinations.

1. Joint reaction forces

**FIGURE 3:**
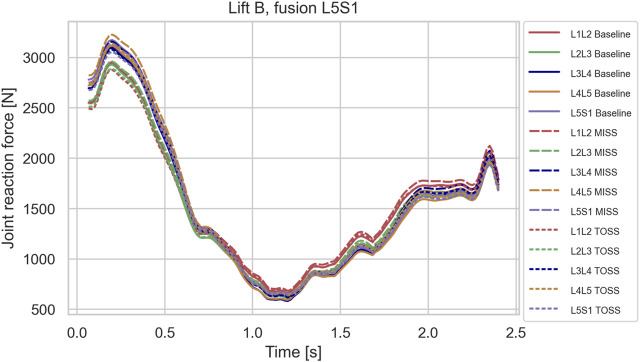
Joint reaction forces in lifting case B for the baseline case and fusion of L5S1 in MISS and TOSS cases respectively. Baseline: solid lines. MISS: long dashes. TOSS: short dashes.

**FIGURE 4:**
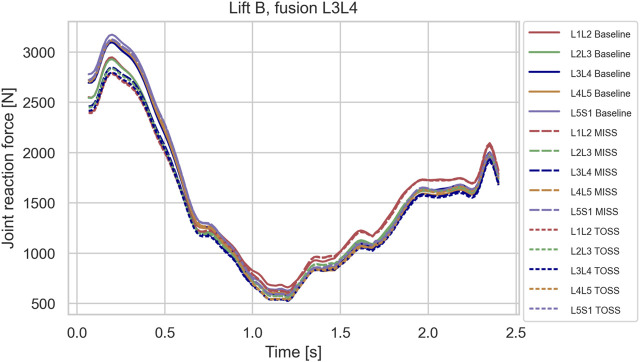
Joint reaction forces in lifting case B for the baseline case and fusion of L3L4 in MISS and TOSS cases respectively. Baseline: solid lines. MISS: long dashes. TOSS: short dashes.

**FIGURE 5:**
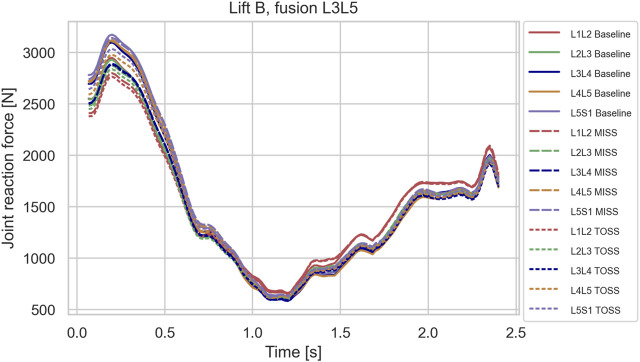
Joint reaction forces in lifting case B for the baseline case and fusion of L3L5 in MISS and TOSS cases respectively. Baseline: solid lines. MISS: long dashes. TOSS: short dashes.

In the sagittal plane lifts, A and B, the maximum lumbar loads over the motion were in the range 2800–3200 N and occurred when picking up the box in the beginning of the motion where spine flexion was at maximum. Since the initial posture was similar for the two lifts, the maximal spinal loads were also similar for these two cases. For these lifts, the differences in joint reaction forces between baseline and the two surgical approaches showed no clear pattern. L4L5 and L3L4, sustained 5–10% higher loads in the initial, flexed posture compared with L2L3 and L1L2, and this applied to baseline as well as both surgical approaches. Figures 3–5 show case B for baseline and selected fusions.

**FIGURE 6:**
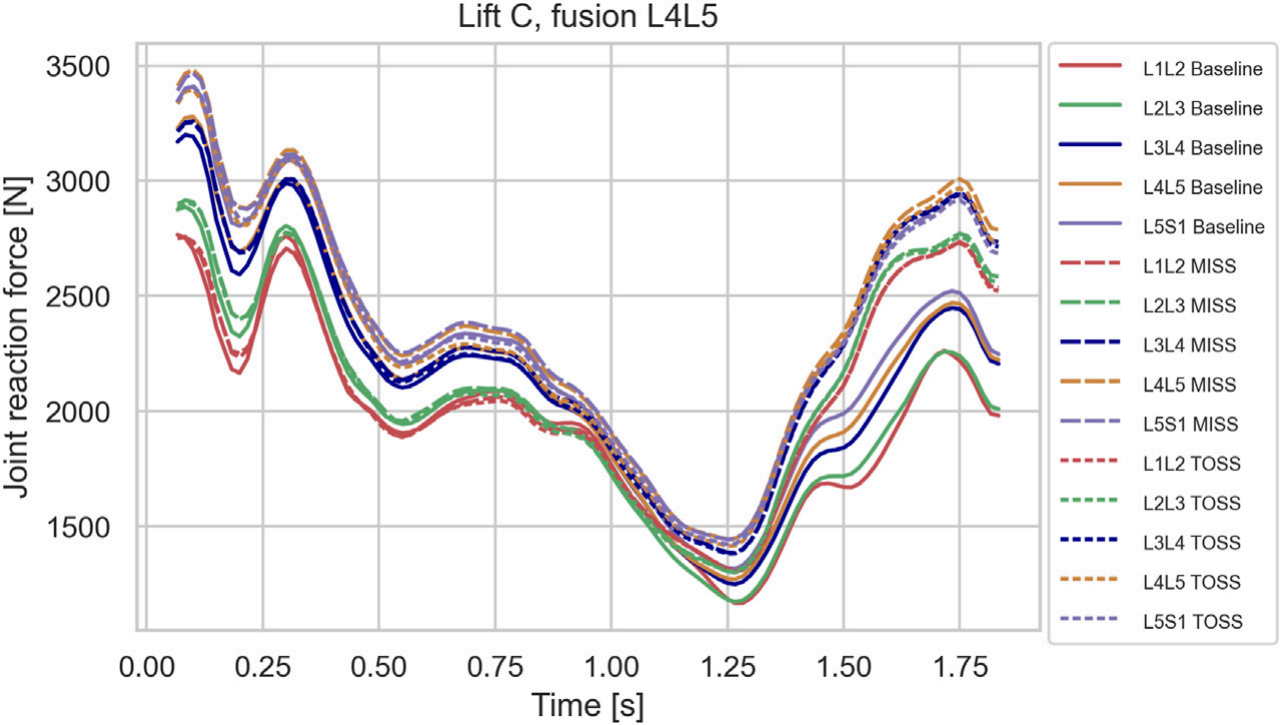
Joint reaction forces in lifting case C for the baseline case and fusion of L4L5 in MISS and TOSS cases respectively. Baseline: solid lines. MISS: long dashes. TOSS: short dashes.

**FIGURE 7:**
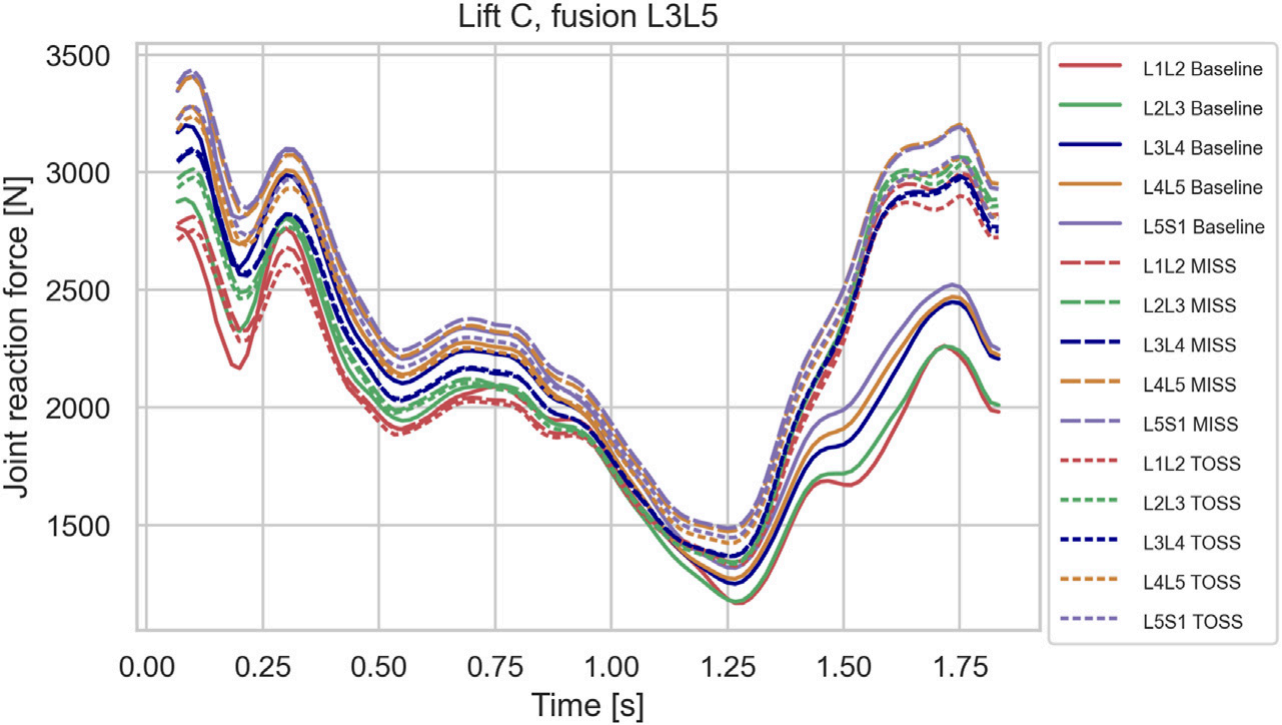
Joint reaction forces in lifting case C for the baseline case and fusion of L3L5 in MISS and TOSS cases respectively. Baseline: solid lines. MISS: long dashes. TOSS: short dashes.

**FIGURE 8:**
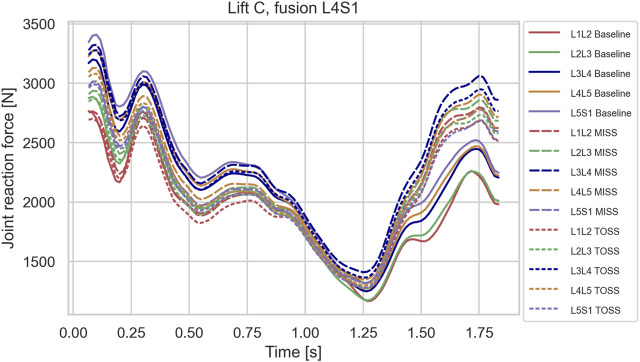
Joint reaction forces in lifting case C for the baseline case and fusion of L4S1 in MISS and TOSS cases respectively. Baseline: solid lines. MISS: long dashes. TOSS: short dashes.

Contrary to the sagittal lifts A and B, the lateral lift, C, did show a clear separation between fused cases and the baseline towards the end of the motion where the subject was reaching laterally to place the box. The load in this posture peaked approximately at *t* = 1.75 s as shown in Figures 6–8, which depict typical examples. Table 2 summarizes the increase of joint load for each fusion case, MISS and TOSS respectively, and each joint relative to the baseline. Spinal loads in the asymmetrical posture of lift C were generally higher in the fused cases than in the baseline case, and more so for the superior fusion sites and for multi-joint fusions. Averaged over all fusion cases and all joints, the TOSS and MISS cases peak at 18 and 21% higher joint force, respectively, in this posture compared with baseline.

2. Muscle loads

**TABLE 2:** Increase of the joint forces relative to baseline in Lift C in the lateral posture near time *t* = 1.75 s for each fusion case.

Joint		Fusion						
		L3L4 (%)	L4L5 (%)	L5S1 (%)	L4S1 (%)	L3L5 (%)	MISS mean	TOSS mean
L1L2	MISS	29	21	7	24	32	23%	
	TOSS	29	21	1	19	28		19%
L2L3	MISS	32	23	9	26	36	25%	
	TOSS	31	22	4	21	34		22%
L3L4	MISS	18	20	10	25	22	19%	
	TOSS	16	20	5	21	21		17%
L4L5	MISS	28	22	10	18	30	21%	
	TOSS	27	20	4	13	24		18%
L5S1	MISS	25	17	1	10	27	16%	
	TOSS	25	16	−4	7	22		13%
Mean		26	20	5	18	28	21%	18%

**FIGURE 9:**
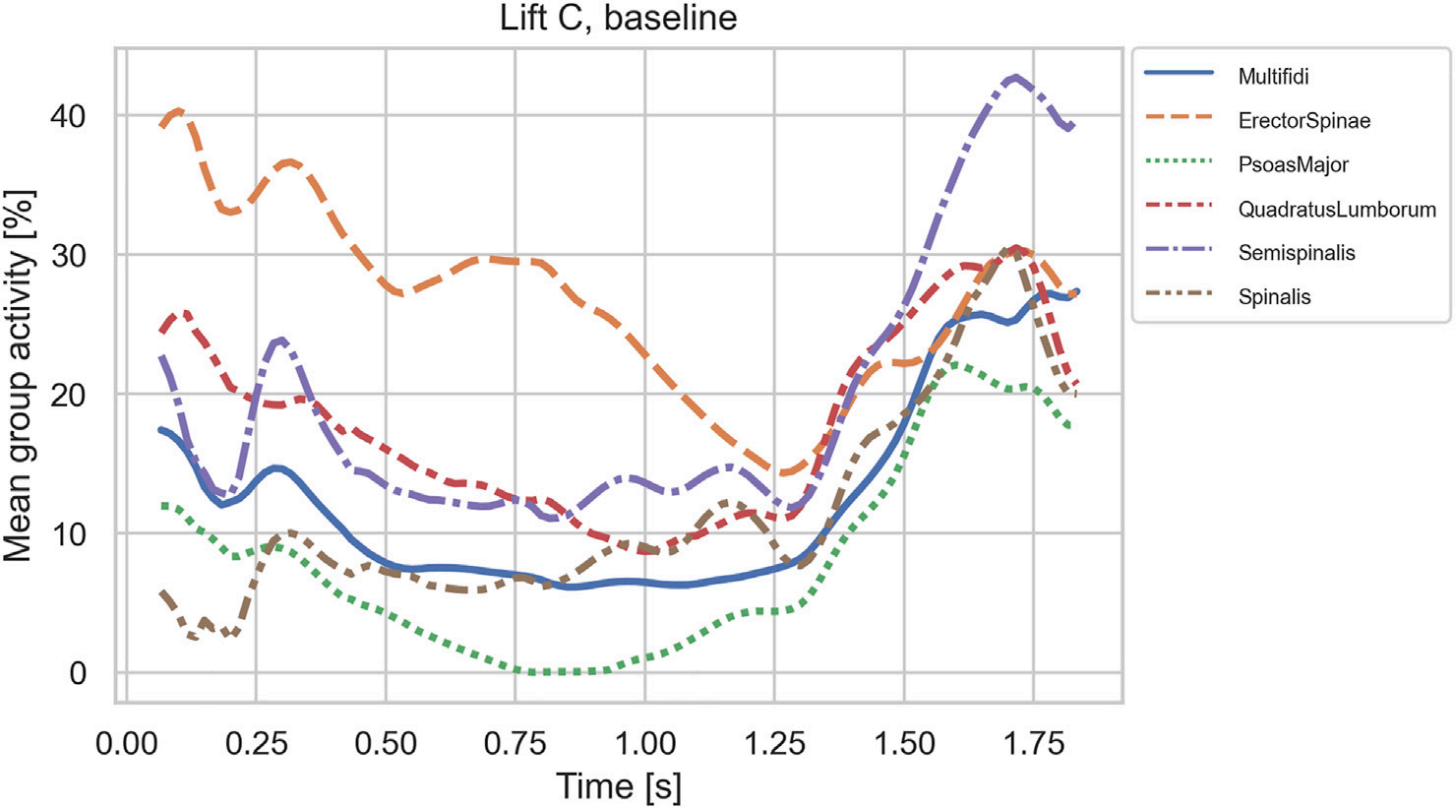
Mean activities of muscle group fascicles in case C on the baseline anatomy.

Figure 9 shows the average activity within each muscle group for lift C in the baseline configuration. The graphs confirm the importance of m. erector spinae for lifting the box from the floor initially, where m. erector spinae fascicles sustained a mean activity of 40%. As the box was moved laterally towards its final position, m. semispinalis fascicles were loaded up to 42% and other muscle groups up to about 30%.

**FIGURE 10:**
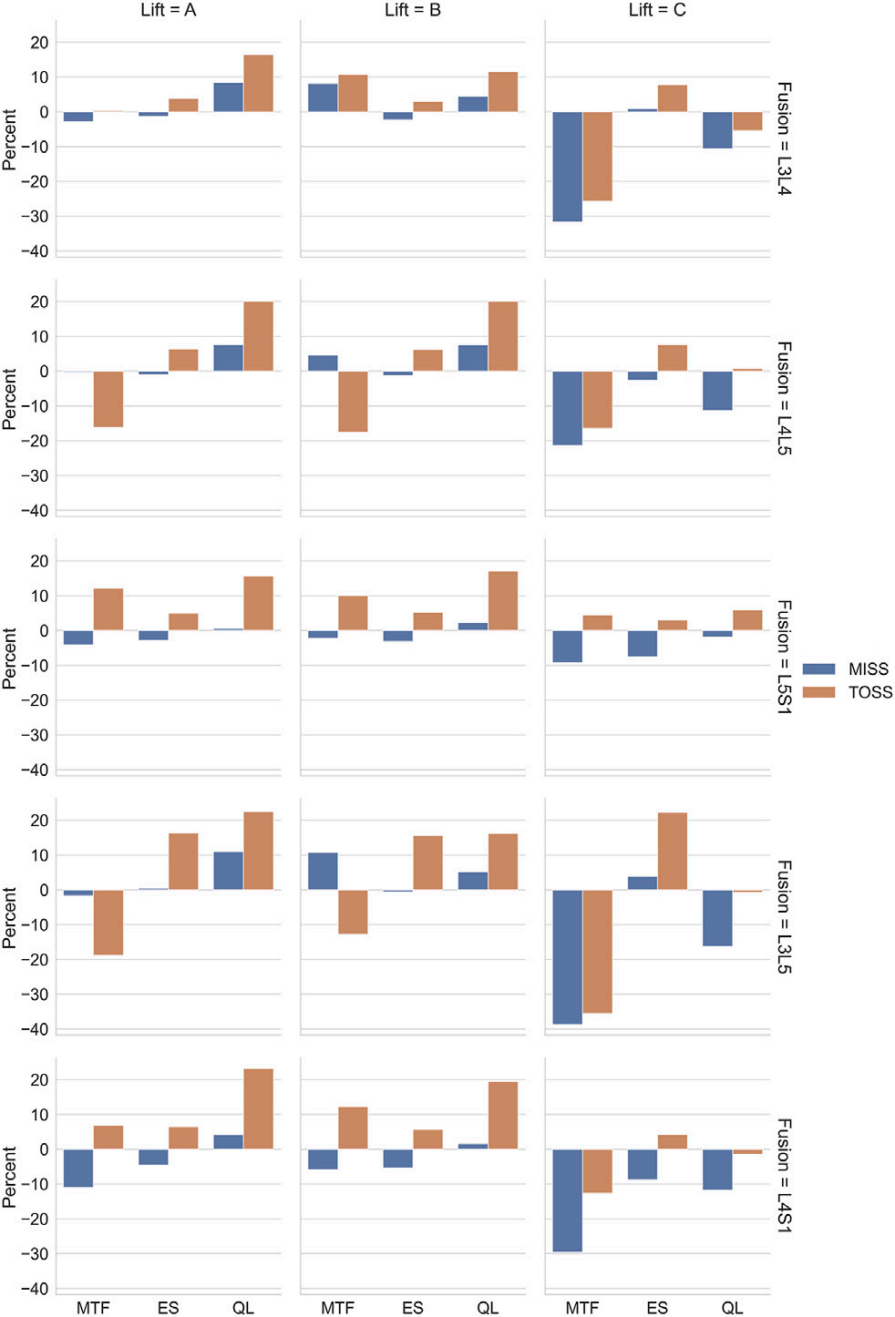
Relative changes of mean loads of different muscle groups for the three lifting cases, A, B and C, as a consequence of different fusions and surgical approaches. Muscle groups: MTF, multifidus, ES, erector spinae, QL, quadratus lumborum.

Relative changes compared with the baseline case of maximum activity levels over the motion for the groups of m. quadratus lumborum (QL), m. erector spinae (ES) and m. multifidus (MTF) for each combination of lifting case (A, B and C), surgical technique (MISS or TOSS) and fusion sites (L3L4, L4L5, L5S1, L3L5 and L4S1) are presented in Figure 10. Each column of plots represents a lifting case, each row represents a fusion case, and the bars are color-coded for MISS and TOSS. Negative values signify an offloading on the average of the muscle group in question by the specified surgery.

The abdominal pressure contributes to the spine extension in the model and therefore works in synergy with the spine extensors. Averaged over the TOSS cases in lift B, the peak abdominal pressure increases by 2.7% compared with the baseline. However, it is primarily the cases involving fusion of the L5S1 joint that contribute, with 8.9% increase for the L4S1 fusion, 6.2% increase for the L5S1 case. The L3L5 fusion for this case reduces the abdominal pressure by −3.2%. The corresponding figures for MISS show a reduction of peak abdominal pressure for all fusions with an average of −1.7%.

## Discussion

Simulated joint forces for sagittal plane lifts (Figures 3–5) are in good agreement with Takahashi et al. (2006), who measured intradiscal pressures for similar lifts of 10 kg in four subjects and calculated disk compression forces from the measured pressures. They found increasing compression with flexion angle up to a maximum about 3 kN. The present model predicts maximum joint forces in the range of 2.8–3.2 kN for lifts A and B.

While it is clinically obvious that MISS reduces the surgical trauma compared with TOSS, mixed results regarding the biomechanical consequences of the two approaches have been reported, as mentioned in the introduction. On the one hand, MISS preserves musculature that is sacrificed by TOSS and, on the other hand, the importance of the resected musculature might diminish, when the joint is fused. We see from Figures 3–5 that spinal joint reaction forces in the sagittal plane lifts A and B were not much influenced neither by the fusion site nor by the surgical approach. This is in agreement with previous clinical and meta studies (Stevens et al., 2006; Min et al., 2009), which have failed to show significant differences between the approaches. Somewhat contrary to our findings, Malakoutian et al. (2016) reported increased adjacent segment loads resulting from muscle weakening and triple-joint fusion in a simulation model. However, this model considered only upright standing and not flexion or lifting.

Figures 6–8, depicting the joint reaction forces in the lateral lift C, show a separation of the curves towards the end of the motion with higher joint reaction forces in the fused cases compared with baseline, regardless of surgical approach. This indicates that the load increase is governed more by the modified kinematics of the partially fused spine than by the altered muscle configuration resulting from the TOSS approach. Previously, finite element models without detailed muscle representations (Park et al., 2015; Rijsbergen et al., 2018) have indicated risk of adjacent disk degeneration following spinal joint fusion. The present model simulates the joint reaction force but not how this force is distributed to the disk. The aforementioned finite element models, on the other hand, take the disk deformation into account, including a possible concentration of stresses caused by the redistributed articulation in adjacent joints. Ideally, the detailed muscle forces and joint articulations simulated in the present model should be transferred to finite element models for computation of tissue stresses, thus exploiting the strengths of both model types.

Figure 10 summarizes the influence of surgical approaches on muscle loads for different fusion cases for the three lifts. Comparing these results column-wise, we see that cases A and B behaved similarly, and case C was different from the other two. The similarity of lifts A and B is because the larger spine loads occur in the more flexed postures, and this part of the movement was common to the two cases. For the sagittal lifts A and B, the changes of muscle activity were larger for the TOSS case compared to MISS, which is not surprising, given that the MISS approach leaves an intact musculature. The load on m. quadratus lumborum increased generally and more for TOSS than for MISS. It is remarkable that the TOSS approach offloaded m. multifidi for the L4L5 and L3L5 fusions. Closer investigation reveals that this was due to elimination of a few multifidi fascicles in the vicinity of the fusion site. The lost extension moment of these fascicles was compensated for by the moment transferred in the fused joint and by fascicles of m. erector spinae, which was also found previously (Bresnahan et al., 2010) for nominal movements with a previous version of the model used in this study. M. erector spinae is the primary spine extensor and has a larger baseline activity, so a given relative increase of its activity in Figure 10 can compensate for a larger relative decrease of m. multifidi fascicles. It is also worth noting that a decrease of the muscular capacity for spine extension due to muscle resection will lead to a larger proportion of the extension moment to be provided by the abdominal pressure. This was shown as an average increase of peak abdominal pressure in the TOSS case. The abdominal pressure causes a distraction force on the vertebrae and can therefore reduce the joint compression forces, provided that the core musculature is capable of producing the additional pressure.

For lift C, m. multifidus was offloaded considerably by all the fusions except L5S1, verifying this muscle group’s role in axial rotation of the spine. The offloading effect was generally larger for MISS than for TOSS. For m. erector spinae, the load increases were smaller for MISS compared to TOSS, and offloading effects were larger for MISS compared to TOSS.

Row-wise comparison in Figure 10 reveals that changes in average muscle activation were larger for higher single joint fusion sites, e.g., L3L4 compared with L5S1, and generally larger for multiple joint fusions, L3L5 and L4S1, than for single joint fusions. Table 2 reveals a similar tendency for the joint loads in lift C. It is remarkable that fusion tended to offload the average m. multifidus activity for MISS and TOSS alike while, as shown in Figures 6–8; Table 2, fusion increased the joint reaction forces in the latter part of the motion. For the TOSS case, the reduced activity in m. multifidi fascicles was compensated for by an asymmetrical addition to m. erector spinae activity. In the MISS case, the joint fusion offloaded some fascicles of the intact musculature at the fusion site, while adjacent fascicles had to exert more force.

The biomechanical conclusions are that spinal fusion regardless of approach type has little influence on spinal joint reaction forces in sagittal plane lifts, but leads to increased loads in lateral lifts regardless of surgical approach. In terms of muscle loads, the spinal fusion can increase loads or offload different muscle groups, but the postoperative loads on the muscular system are generally smaller for MISS than for TOSS approaches.

In a clinical perspective, the results add biomechanical support for the case of MISS versus TOSS, but perhaps less than expected when considering the changes of muscle configuration involved in TOSS. Individuals with fused joints, regardless of the approach, should be advised to be particularly careful with asymmetrical lifts. Although this recommendation is accepted ergonomics practice, there appears to be biomechanical reasons to emphasize the recommendation to this patient group.

Human biomechanics is quite complicated, and simulation results should be used with caution. Most of the output variables in the present investigation are infeasible to measure *in-situ* on test subjects, and this challenge represents simultaneously the motivation for using models and the difficulty in terms of validating them. Known model limitations should therefore be borne in mind: The present spine model is limited to the lumbar and cervical sections, while the thoracic section and rib cage are considered as a single, rigid body. The model includes the extension effect of the abdominal pressure and its connection with m. transversus abdominis activation, but its implementation does not comprise the complexities of the diaphragm and pelvic floor. These shortcomings are the subject of ongoing research, and the results of this paper should be reevaluated continuously as models with higher fidelity become available.

It is a limitation of the study that input data were collected from a single, able-bodied individual performing only three different but related tasks. Generalization to patient populations would require data that account for variation in terms of anthropometry, motion patterns, gender, age and possibly other variables. The three lifting tasks are hardly representative for activities-of-daily living in general, and the finding that joint load tendencies are different for the two types of lifts, i.e., A/B versus C, indicates the necessity to perform biomechanical evaluation on a larger variety of activities-of-daily-living. Research to identify such a representative set of activities, against which biomechanical evaluation of spinal surgery can be performed, would be a valuable contribution towards *in-silico* models with clinical fidelity.

## Data Availability

The raw data supporting the conclusion of this article will be made available by the authors, without undue reservation.
